# Remarks on the Use of the Letheon; with Cases

**Published:** 1848-04

**Authors:** J. F. Sanford

**Affiliations:** Farmington, Iowa


Art. IV.—*Remarks on the Use of the Letheon, with Cases*: By J. F. Sanford, M.D., of Farmington, Iowa.

There has been perhaps no discovery in the Medical Sciences, either in ancient or modern times, the importance of which has been so great, as that of Letheon, and none which has been established upon a basis so incontrovertible, or the vast utility of which has been so speedily acknowledged. This result, so satisfactory to the feelings, and so honorable to the genius of the discoverer, is mainly attributable to the alacrity with which the mass of the medical profession throughout the world, have subjected it to the test of experience, and the facility with which its intrinsic merits may be satisfactorily demonstrated.

A truth in philosophy, and particularly medical philosophy, possesses an importance in exact proportion to its agency in forwarding the great ends and objects of science—the mitigation of the "thousand ills that flesh is heir to." And when such is the certain effect of its establishment, too much experience cannot be adduced in its support, and it cannot be rendered too familiar to the common mind. Influenced by these reflections—notwithstanding that the sanction of the eminent and scientific in our profession, has placed the utility of the Letheon beyond dispute, I have resolved to communicate to the profession the result of my experience in its use, hoping, that, if I present nothing that is new, it will at least interest some to hear that the benefits of this great discovery are not confined to the cities and hospitals of the eastern and middle states.

The first case in which I applied the ether, was that of a young lady, thirteen years of age, of nervous temperament and delicate constitution, who, two months previous to my seeing her, had suffered a severe fracture of the first and second phalanges of the middle finger of the right hand. Various applications were made to the wound at the time by the parents, who did not regard it as a serious matter; but severe inflammation of the neighboring tissues supervening in a few days, a quack living in the vicinity was called in, who made use of numerous stimulating applications, to arrest as he termed it, the mortification, and repress the fungous growths. The inflammation continued, however, to increase in intensity from day to day, and despairing of aid from "Doctors," the parents dismissed their attendant, and began the application of emollient poultices. Under this treatment the surrounding inflammation began to decline, and in two weeks, had subsided except in the wound, which presented a very irritable appearance and was exceedingly sensitive and painful; finally the soft parts began to slough away, the first phalanx in a fractured state was thrown off, and the process of inflammation and sloughing was gradually extending to the hand. At this stage of the case, I was called on to amputate the finger.

When I arrived at the house, I found my patient sitting on the bed-side, sustaining the diseased member in an elevated position—the only way in which she could have any ease—and sobbing aloud in anticipation of the pain of the operation. She was very much emaciated and exceedingly irritable. A brief examination convinced me, that unless some means were used to prevent it, severe constitutional effects would follow an operation upon so delicate a subject; I suggested the ether to a medical friend who accompanied me, who, although he had never witnessed its application, was willing to make the trial. After every thing was arranged, and the proper directions given to the patient and friends, I saturated a linen handkerchief with sulphuric ether, and partially enclosing it in a piece of silk, applied it to the nostrils and mouth of the patient; in three minutes, without evincing the slightest evidence of exhileration, she was perfectly etherized—lying in a relaxed condition—the eyes turned up, and an expression of pleasure upon the countenance. The operation was at once proceeded to—the diseased finger was firmly grasped—a semi-circular sweep of the knife, half an inch anterior to the metacarpo-phalangeal articulation was made—transfixion effected, and a flap formed anteriorly—disarticulation completed the operation, during which, notwithstanding the exquisitely sensitive condition of the parts, the patient manifested not the slightest evidence of pain. A few moments afterwards consciousness and sensibility returned, but no pain or other disagreeable symptom was complained of, and from this time my patient rapidly improved in strength and flesh. The wound healed kindly by the first intention—no fever or excitement occurred during the progress of recovery, and all things went on well.

The next case in which I had occasion to use ether, was that of a lady, æt 32, of nervo-bilious temperament, who had suffered for three years from an irritable tumor of the right mamma, about the size of a hen's egg. Without any appreciable cause the tumor began to grow three years since, and in that time, attained the size mentioned above. Usually, it was hard, dense, insensible, and slightly adherent to the surrounding parts, but occasionally, from cold or other causes, would become sensitive and painful, involving the surrounding tissues in inflammatory action and exciting great constitutional disturbance. At these periods it was also considerably enlarged. Being consulted during one of these paroxysms, I treated it upon the antiphlogistic plan, which soon relieved the patient, and after the inflammation subsided I was requested to take charge of the case, with a view to effect a permanent cure. I of course advised the removal of the tumor with the knife; this was consented to, and I proceeded to the operation, after succeeding in the most satisfactory manner in etherizing the patient. During the operation there was not the slightest indication of pain. My patient recovered rapidly without a bad symptom—the wound healing kindly; and since the operation, (20th August, 1847,) she has had no pain in the breast—a longer exemption than she has enjoyed since the appearance of the tumor.

On the 16th day of December, 1847, I amputated the right leg of Mr. M., æt 54, after treating it several months, for a disease of the ankle joint—Synovial degeneration with caries of the astragalus and tibia. As is my custom, I had given him the ether a few days before, to ascertain the degree of susceptibility of his system to its influence, and to familiarize him to its administration, and found it to do well. On the day above mentioned, I proceeded in the presence of several Physicians to etherize M. and perform the operation. Perfect etherization was readily induced—the patient taking the vapor from a tin vessel about the size of a diploma-case, with a spout projecting a few inches from the top, and flattened at the end to be easily received into the mouth. During the operation, which occupied about two minutes, not a muscle was moved, the patient being entirely insensible and unconscious of what was going on; when the influence of the vapor passed off, he was perfectly tranquil, and expressed his surprise that he had suffered no pain. This patient had the speediest, and most happy recovery from an amputation I have ever known. There was not the slightest fever, pain, increase of temperature in the limb, undue inflammation in the stump, or any disagreeable complication whatever. His health, which had become greatly impaired, began to improve from the day of the operation, and four weeks afterwards he was going about on a wooden leg, attending to the duties of his farm.

Since that time, (16th Dec.) I have performed a number of minor operations under the influence of ether, and with the same success; and I am most firmly convinced of its safety and efficiency as an anæsthetic agent in all cases.

It will be observed that necessity caused me to resort to the ether, in the first case detailed. This was the first person I ever saw under its influence; I used the common sulphuric ether of the shops, and succeeded fully, notwithstanding that my inexperience and other circumstances were against me. The only increased facility I have enjoyed in subsequent operations, has been the use of the instrument mentioned above, which renders the exhibition of the vapor much more convenient: sometimes, however, my patients become a little intractable—refuse to inhale from it, and I am compelled to resort to my first expedient; in this way I can coerce the inhalation until the influence is fully induced. In no case have I been unsuccessful in its use, except in a single instance in which I applied it in obedience to the patient's importunity, and against my own judgment. This person had taken, an hour or two before, a free draught of spirits, and when he had inhaled the ether a short time, became excited and unmanageable. A few hours after, he took the vapor, and was operated upon without suffering any pain.

Is it then, true, as the experience of some seems to establish, that this article is adapted to only a certain class of cases? and that particular temperaments and conditions are alone susceptible to its favorable influence? Unhesitatingly, I would answer this question in the negative, and adduce an experience, varied, and somewhat extensive for a private practitioner, in defence of the position. I am persuaded, and feel anxious to attract the attention of the profession to the fact, that every case of partial or complete failure in any temperament or condition, is entirely attributable to the timidity of the operator or the patient. The majority of practitioners proceed to its exhibition, lacking full confidence in its powers, or having undue fears of its deleterious effects, and become easily discouraged. Patients take it with an excited imagination—mingled diffidence and fear; the excitement incident to the first stage of etherization is induced—they become a little unmanageable, and the attempt is abandoned.

When this state of things occurs in my practice, I usually lay aside the inhaling apparatus, as above remarked, and resort to the plan adopted in the first case. A moment's perseverance only is required to insure tranquillity, and complete etherization, and no bad effect has ever followed its administration in this way, to any of my patients. They universally express a feeling of comfort and ease ten minutes after the effect passes off, being exempt even, to a great extent, from that burning pain in the wound, always complained of after operations performed without the ether. I have not noticed that the action of the heart is materially affected during the influence, or that the functions of the respiratory organs suffer any derangement.

My experience, then, justifies the following deductions:

1st. Operations performed upon persons under the complete influence of the ether, do not cause the shock to the nervous system and depression of its energies, which necessarily follow operations performed under other circumstances.

2d. This shock and depression of the nervous system being averted, the consequent and corresponding reaction and excitement do not occur, and fever, inflammation, and their concomitant evils are not to be feared.

3d. The careful and cautious administration of ether is not attended or followed by any bad effects; and patients subjected to its influence during capital and other operations, *cæteris paribus*, enjoy greatly increased chances of recovery.

4th. It is applicable to every variety of cases, except perhaps where there is organic lesion of the cerebral tissues. In these cases I have had no experience.

These conclusions may have been established by other practitioners; if so, I am not aware of it. Living remote from the best sources of information I am not as enlightened upon this subject as thousands of my professional bretheren; but almost every article I have read relating to this agent, expresses doubts as to its general application and complete efficacy. It is against this sentiment that I desire to raise my voice; and feeling, as far as my experience justifies me in doing, the utmost confidence in, and the profoundest admiration of, this splendid discovery, I shall express the hope that the time is not far distant, when the force of truth in its favor, like the waters of the celebrated river of Tartarus whose name it bears, shall obliviate all prejudice and opposition to a discovery, so highly calculated to alleviate human suffering.

February 6, 1848.

